# A Core Genome Multilocus Sequence Typing Scheme for Enterococcus faecalis

**DOI:** 10.1128/JCM.01686-18

**Published:** 2019-02-27

**Authors:** Bernd Neumann, Karola Prior, Jennifer K. Bender, Dag Harmsen, Ingo Klare, Stephan Fuchs, Astrid Bethe, Daniela Zühlke, André Göhler, Stefan Schwarz, Kirsten Schaffer, Katharina Riedel, Lothar H. Wieler, Guido Werner

**Affiliations:** a Robert Koch Institute, Division of Nosocomial Pathogens and Antibiotic Resistances, Wernigerode, Germany; b Department of Periodontology and Restorative Dentistry, University Hospital Muenster, Muenster, Germany; c Institute of Microbiology and Epizootics, Centre for Infection Medicine, Freie Universität Berlin, Berlin, Germany; d Department for Microbial Physiology and Molecular Biology, Institute of Microbiology, University of Greifswald, Greifswald, Germany; e Friedrich Loeffler Institute of Medical Microbiology, University Medicine Greifswald, Greifswald, Germany; f Department of Microbiology, St. Vincent’s University Hospital and School of Medicine and Medical Science, University College Dublin, Dublin, Ireland; g Robert Koch Institute, Berlin, Germany; University of Iowa College of Medicine

**Keywords:** *Enterococcus faecalis*, core genome MLST, molecular surveillance, molecular typing, whole-genome sequencing

## Abstract

Among enterococci, Enterococcus faecalis occurs ubiquitously, with the highest incidence of human and animal infections. The high genetic plasticity of E. faecalis complicates both molecular investigations and phylogenetic analyses. Whole-genome sequencing (WGS) enables unraveling of epidemiological linkages and putative transmission events between humans, animals, and food. Core genome multilocus sequence typing (cgMLST) aims to combine the discriminatory power of classical multilocus sequence typing (MLST) with the extensive genetic data obtained by WGS. By sequencing a representative collection of 146 E. faecalis strains isolated from hospital outbreaks, food, animals, and colonization of healthy human individuals, we established a novel cgMLST scheme with 1,972 gene targets within the Ridom SeqSphere^+^ software. To test the E. faecalis cgMLST scheme and assess the typing performance, different collections comprising environmental and bacteremia isolates, as well as all publicly available genome sequences from the NCBI and SRA databases, were analyzed. In more than 98.6% of the tested genomes, >95% good cgMLST target genes were detected (mean, 99.2% target genes). Our genotyping results not only corroborate the known epidemiological background of the isolates but exceed previous typing resolution. In conclusion, we have created a powerful typing scheme, hence providing an international standardized nomenclature that is suitable for surveillance approaches in various sectors, linking public health, veterinary public health, and food safety in a true One Health fashion.

## Abstract (precis)

Among enterococci, Enterococcus faecalis occurs ubiquitously, with the highest incidence of human and animal infections. The high genetic plasticity of E. faecalis complicates both molecular investigations and phylogenetic analyses.

## INTRODUCTION

Enterococcus faecalis is a ubiquitous environmental and opportunistic Gram-positive bacterium that colonizes the gastrointestinal tract of humans and various animals (1). E. faecalis is also used in commercial probiotic products and is an important ingredient in food production, such as in fermented sausages or cheese from raw milk (2–5). Among members of the genus Enterococcus, this species shows the highest incidence for human and animal infections and, in addition, is discussed as a zoonotic pathogen (5–7). E. faecalis has emerged as a major nosocomial pathogen worldwide, causing bloodstream infections mainly in immunocompromised humans. Furthermore, it is responsible for endocarditis and urinary tract infections acquired on an outpatient basis, as well as for mastitis in dairy cattle (8–10). Due to their ability to survive even harsh environmental conditions, enterococci pose an immense hygienic challenge to clinical settings (11–13). Most importantly, treatments of enterococcal infections are limited to only a few therapeutic agents, since enterococci exhibit multiple intrinsic resistances and are known to easily acquire additional resistance determinants (14, 15). Hence, multidrug-resistant enterococci, especially vancomycin-resistant enterococci (VRE), are a burden for hospitals worldwide (16, 17).

To validate epidemiological linkages and disclose possible transmission events among humans, animals, food, and the environment, discriminatory high-resolution typing is of the utmost importance. The high genetic plasticity of E. faecalis is the result of wide exchanges of genetic material, which in turn complicate molecular investigations (18). In contrast to the second most important Enterococcus species in hospital settings, Enterococcus faecium, for which hospital-adapted lineages have already been identified, most E. faecalis genotypes do not show extended host or context specificity (19). For instance, Buhnik-Rosenblau et al. demonstrated, on the basis of multilocus sequence typing (MLST), that no specific genetic groups could be assigned to a colonization- or infection-associated origin (5). Nevertheless, other studies have described at least one hospital-associated E. faecalis lineage, to which sequence type 6 (ST6) belongs (20–23).

Classical MLST relies on PCR amplification and the sequencing of seven housekeeping genes that are distributed over the bacterial chromosome (23, 24). Classical MLST provides an international and expandable typing nomenclature, but the method provides only a moderate typing resolution. Typing by macrorestriction analysis via pulsed-field gel electrophoresis (PFGE) is limited in its discriminatory power, and it sometimes displays discrepancies with whole-genome sequencing (WGS)-based investigations (25, 26). Since next-generation sequencing (NGS) enables high-throughput analyses of entire bacterial genomes at an affordable cost, it has quickly become indispensable for performing population and outbreak analyses. However, appropriate bioinformatics tools are necessary as a prerequisite for handling and interpreting sequence data (25, 27, 28). Core genome MLST (cgMLST) aims to combine the discriminatory power of classical MLST with the extensive genetic data derived from WGS (29, 30). Exploiting hundreds of gene targets of the entire bacterial genome, thereby providing maximum resolution for multiple research and surveillance analyses (31, 32), is one of the greatest advantages of the cgMLST scheme. Implementing this scheme in the SeqSphere^+^ software (Ridom GmbH, Münster, Germany) allows the definition and curation of an international and standardized nomenclature, which has successfully been developed for other meaningful pathogens, such as Staphylococcus aureus, Listeria monocytogenes, and Enterococcus faecium (29, 33, 34).

In this study, we generated a powerful typing scheme for E. faecalis using the SeqSphere^+^ software, hence providing an international standardized nomenclature that is suitable for surveillance approaches, outbreak investigations, and phylogenetic analyses.

## MATERIALS AND METHODS

### Isolate collection.

To define the scheme, a total of 146 E. faecalis isolates (Table S1) were selected from the strain collection of the German National Reference Centre (NRC) for Staphylococci and Enterococci. The collection comprises isolates from different sources (hospital associated, human colonization, animal, and food) sent to the NRC over a period of 20 years. Hospital-associated strains were isolated from blood (bacteremia or endocarditis), urine, and wounds. Isolates designated human colonization strains were obtained from stool specimens or rectal swabs as part of patient screening. The animal-associated strains were isolated from dairy cattle, pig, and poultry specimens that included healthy animals, confirmed infections (mastitis), and food (meat). The selection of isolates also included VRE of the most abundant vancomycin-resistant genotypes, *vanA* (*n* = 24) and *vanB* (*n* = 19). For calibration of the novel cgMLST scheme, a subset of closely related E. faecalis strains was included. Of these, 27 isolates that were assigned to 10 PFGE types were obtained from an intestinal colonization screening (ICS) program of healthy, nonhospitalized families in Germany. Also, 23 isolates from 4 putative hospital transmission (PHT) events and forming 4 PFGE types (>82% identity) were selected.

Furthermore, two additional isolate collections were compiled to test the newly defined cgMLST scheme (Table S1). First, a collection of 14 E. faecalis isolates from blood cultures, representing all E. faecalis blood culture isolates sent to the NRC in the years 2015 to 2017, was sequenced and analyzed. Second, a total of 21 environmental samples, isolated from hospital wastewater and the follow-up treatment stages, were analyzed.

### DNA extraction, whole-genome sequencing, and *de novo* assembly.

Bacterial strains were cultivated overnight in brain heart infusion broth at 37°C. DNA was extracted using a DNeasy blood and tissue kit according to the protocol of the manufacturer (Qiagen, Hilden, Germany). The Qubit double-stranded DNA (dsDNA) high-sensitivity (HS) assay kit (Invitrogen/Thermo Fisher Scientific, Karlsruhe, Germany) was used for DNA quantification. A total of 1 ng of extracted DNA was employed for library preparation using the Nextera XT DNA library prep kit according to the manufacturer’s instructions (Illumina, San Diego, CA). Whole-genome sequencing was accomplished using short-read (2 × 300-bp) paired-end sequencing provided by MiSeq (*n* = 109) and HiSeq (*n* = 72) instruments (Illumina, San Diego, CA) as described elsewhere (15). The resulting raw reads were assembled *de novo* using SPAdes (v. 3.9.0, default parameters) (35), which is also integrated into the Linux version of the SeqSphere^+^ software (v. 5.0.0; Ridom GmbH, Münster, Germany). The assembled sequence data were used for extraction of STs and further downstream analyses.

### Single nucleotide polymorphism-based mapping.

For comparative purposes and validation of the cgMLST scheme, the sequence data obtained were also used for a single-nucleotide polymorphism (SNP) mapping approach. To this end, raw reads were trimmed using Trimmomatic (v. 0.32; default parameters with sliding window set to 4:15) (36), and resulting paired-end reads were aligned to the reference sequence of E. faecalis strain OG1RF (GenBank accession no. NC_017316) using BWA-SW (v. 0.7.13-r1126; default parameters) (37). Subsequent variant calling was performed using VarScan (v. 2.3; default parameters) (38). E. faecalis is highly recombinogenic; therefore, SNPs located within a distance of 300 bp or less to each other were excluded using SNPfilter (v. 3.2.3; exclusion distance [*d*] = 300) (39). Maximum likelihood phylogenetic trees were calculated on the basis of retained SNPs using PhyML with a general time reversible (GTR) nucleotide model (v. 3.0; bootstrap, 1,000 permutations) (40). Phylogenetic trees were visualized by applying iTOL (v. 4.2.3; https://itol.embl.de/) (41).

### Target scheme definition.

The finished and publicly available genome of E. faecalis OG1RF (version 1, GenBank accession no. NC_017316.1) was selected as the “seed genome” for the cgMLST scheme definition. Using the cgMLST Target Definer tool (v. 1.5 with default parameters) of the Ridom SeqSphere^+^ v. 4.0.9 software, a rapid local *ad hoc* cgMLST scheme was defined that contained all genes of the reference genome that were not homologous, did not contain internal stop codons, and did not overlap other genes. All other 11 publicly accessible and finished E. faecalis genome sequences (as of 12 May 2017) (Table S1), together with the 146-shotgun genome collection of this study (see above), were selected as query genomes to generate a cgMLST task template applicable to E. faecalis strains of various origins. Only targets from the *ad hoc* scheme that were present in ≥95% of all query genomes were accepted as cgMLST targets. This parameter was chosen due to the genetic plasticity and the diverse population structure of E. faecalis and to ensure a typing scheme suitable for isolates of various origins. The resulting compilation of target genes (Data Set S1) was defined as the core genome genes and used for the subsequent typing scheme.

### Evaluation of the cgMLST target gene set.

All publicly available genome sequences from the NCBI and NCBI Sequence Read Archive (SRA) databases (as of 30 August 2017) were used to evaluate the cgMLST scheme. In total, 526 NCBI genomes were downloaded and analyzed in SeqSphere^+^. To ensure a defined and quality-controlled data set for the evaluation process, a thorough manual filtering process was performed. First, all genomes used to create the cgMLST task template were removed from the evaluation data set (*n* = 514 after filtering). Next, all data sets sequenced with Ion Torrent or 454 technology were excluded because of their increased tendency to contain homopolymer errors (*n* = 502). Afterwards, and in order to ensure sufficient quality of the sequence data used, all data records for which no MLST could be assigned by SeqSphere^+^ were filtered out (*n* = 488). Finally, the data sets were searched for duplicates, since prominent or culture collection strains are often overrepresented and could thus induce a bias in the evaluation data set (*n* = 481). Genome sequences from the SRA were automatically filtered with SeqSphere^+^ by (i) Illumina data, (ii) SRA replicate filtering (technical replicates of one SRA sample were identified and the biggest data set kept), and (iii) strain name filtering, to detect strains submitted more than once, e.g., by different submitters (the largest data set was selected). After filtering, 984 of 2,093 genome sequences met these criteria. One single-genome sequence was unavailable, so a total of 983 SRA sequences were finally downloaded for scheme evaluation. All data sets were assembled with SPAdes (v. 3.9.0 implemented in SeqSphere^+^ with default settings [“–careful” option turned off]) and analyzed with SeqSphere^+^. Most of the manually processed filter criteria used for the NCBI data were implemented in the SRA download and automatically processed. Therefore, only SRA data duplicates of the seed and query genomes (*n* = 978), as well as low-quality data, i.e., data sets to which no MLST could be assigned, had to be removed by manual filtering, leaving 849 data sets of the NCBI-SRA collection. A final manual filtering step omitted 330 genome data duplicates derived from merging the SRA and NCBI sequences (NCBI data were kept while SRA data were removed). Finally, 1,000 data sets were retained for cgMLST evaluation.

### cgMLST-based analysis.

The environmental strains, NRC and NCBI strains, and all isolates used to define the novel cgMLST were analyzed using the cgMLST scheme to validate the applicability of the scheme and review the collection for its representativeness of the E. faecalis population structure. The software defined individual allelic profiles at the strain level. These profiles were used to construct minimum spanning trees (MST) in SeqSphere^+^ for the entire strain collection by pairwise ignoring missing values. Furthermore, phylogenetic trees were calculated using the neighbor-joining algorithm with default parameters implemented in the Ridom SeqSphere^+^ software. Trees were visualized using iTOL (v. 4.2.3) (41).

### Determination of cluster distance.

To determine allelic distances between closely related isolates, the calibration subsets of isolates obtained from the ICS program (*n* = 27) and from PHT events (*n* = 23) were used. Detailed knowledge about the epidemiological background of these subsets further strengthened the selection chosen for calibration. The threshold for cluster distance determination was set based on the results of the cgMLST-based MST using the allelic differences between highly identical isolates compared with allelic differences from or between nonrelated isolates. For validation purposes, the threshold derived was compared with results of the SNP-based mapping analyses.

### Comparison of phylogenetic trees.

The resulting phylogenetic trees from the SNP- and cgMLST-based approaches to the collection of 146 isolates were used to compare the concordance of the two methods. The phylogenies were checked for similarities and differences regarding isolate clustering. In addition, using normalized absolute differences of patristic distances of isolate pairs obtained by the respective phylogenetic analyses, an isolate-by-isolate comparison was performed. It was necessary to multiply the difference values by 1 × 10^9^, followed by taking the logarithm (basis = 2), to generate distance values suitable for comparing the two approaches. These calculations resulted in values that were used as a measure of the grade of concordance, which then was visualized by heat maps using iTOL (v. 4.2.3) (41). In doing so, the logarithm compressed the data distribution. To mitigate this effect in the visualization, the median of the values was calculated and used as the mean value of the color scale.

### FastTree analyses with available sequence types.

A maximum-likelihood phylogenetic tree was generated by FastTree 2 (v. 2.1.7, Jukes-Cantor+CAT model) using aligned concatenated sequence data of 852 MLST sequence types (as of 25 January 2018) obtained from PubMLST (www.pubmlst.org) (42, 43). The resulting phylogenetic tree was visualized by applying iTOL (v. 4.2.3) (41).

### Accession number(s).

Raw reads of all sequenced E. faecalis isolates of this study (*n* = 181) were submitted to the Sequence Read Archive database of the National Center for Biotechnology Information and are available under accession no. SRP156712.

## RESULTS

### Diversity and suitability of the strain collection for scheme definition.

MLST analyses revealed 41 different STs present in the strain collection of 146 isolates, 7 of which were new STs submitted to the PubMLST database (42). Considering the diverse origin of the isolates (hospital associated, human colonization, animal, and food) and the sampling period of more than 20 years, the collection provided a deep insight into the population of E. faecalis.

**FIG 1:**
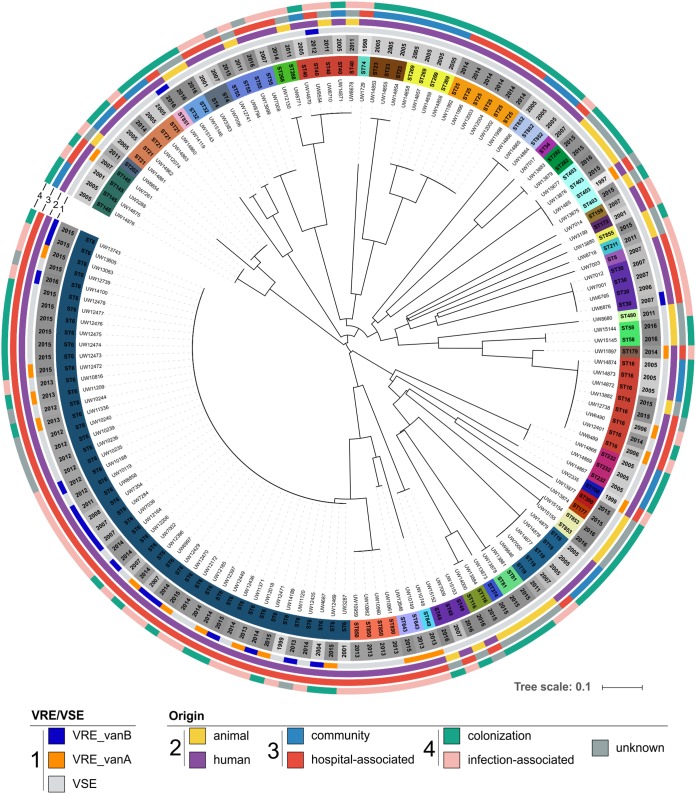
Isolate collection for establishing the E. faecalis cgMLST scheme (*n* = 146). The phylogenetic tree is based on MLST analyses and was calculated with SeqSphere^+^. Sequence types (STs) were added and color-coded in the inner circle. Year of strain isolation was added and shaded as a gray circle (light gray to dark gray). Vancomycin resistance and the *van* genotype were also added as color-coded circles (see key, “VRE/VSE”). In the three outer circles (2 to 4), the origin of isolation was depicted (see key, “Origin”). Circle 2 depicts whether an isolate is of human (purple) or animal (yellow) origin. Circle 3 represents the origin of human isolates (blue, community; red, hospital associated). The outer circle, 4, shows whether an isolate was obtained from colonization (green) or was infection associated (pink). For some isolates, the data were not valid or were unknown (dark gray). Visualization was realized using iTOL.

To visualize the genetic relation between isolates of the collection, a phylogenetic tree based on MLST genes was calculated using the Ridom SeqSphere^+^ software (Fig. 1). The MLST tree was annotated with available metadata of all isolates, in particular, ST, origin, vancomycin resistance, and year of isolation. In due consideration of the importance of the human- and clinical-associated lineage ST6, which also included most of the VRE, this genotype was more frequently represented in the collection (*n* = 47, ∼32%).

**FIG 2:**
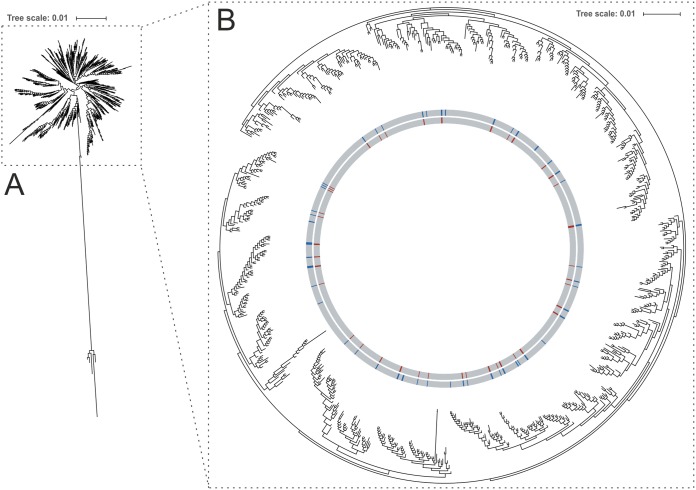
The phylogenetic tree of 852 sequence types (STs) represents the distribution of STs used for cgMLST scheme development within the global ST population of E. faecalis and was generated using FastTree 2. (A) A radial overview and (B) a circular presentation of the main part of the tree are shown. STs represented in the strain collection for scheme definition are color coded in red. Blue color codes represent all STs available for this study. Visualization was realized using iTOL.

To gain an insight into the representativeness of the strain collection in the context of all available STs, a maximum-likelihood phylogenetic tree based on sequences of 852 STs was calculated using FastTree 2 (Fig. 2). The resulting tree showed clustering of available sequence types, with each single leaf representing one ST. As can be seen in Fig. 2, the selected strains were distributed over the entire tree and covered almost every branch, indicating that the strain collection reflects an extensive and diverse selection of the E. faecalis ST-based population structure (Fig. 2B). Only an outlier group of five different STs was not represented in the isolate collection used to define the cgMLST scheme (Fig. 2A).

### Development and evaluation of the E. faecalis cgMLST scheme.

Based on the E. faecalis strain OG1RF reference genome (2,636 genes with coding DNA sequences), a preliminary reference task template was generated that retained a total of 2,385 target genes after thorough filtering. Using all 11 finished genome sequences from NCBI (as of 15 January 2017) and the 146 genome sequences of our strain collection as penetration query genomes, a cgMLST scheme of 1,972 target genes was defined, covering 67.9% of the seed genome (Data set S1). All finally assigned cgMLST targets were present in ≥95% of all query genomes.

For evaluation purposes, 1,000 genome sequences from the NCBI and SRA databases (as of 30 August 2017) were downloaded, thoroughly filtered, and finally analyzed using the E. faecalis cgMLST scheme. At least 95% good cgMLST target genes were found in more than 98.6% of the genome sequences (mean, 99.2% good target genes). In addition, the 146 query genome sequences of the isolate collection were also used to analyze the performance of the newly defined cgMLST scheme. Query genome analysis resulted in a mean of 99.6% good targets detected (ranging from 96.4% to 100%) (Table S1; “% good cgMLST targets” column).

To determine cluster distances, a subset of 50 isolates with detailed epidemiological data (ICS, PHT) was used. The ICS isolates belonged to 10 distinct groups determined by PFGE analyses and comprised 9 different STs (Fig. S1). Relatedness was further supported by the results from the SNP-based analysis, as these groups exhibit 0 to 1 SNP differences (Data Set S2). The isolates from PHT also belonged to four distinct PFGE groups, comprised three different STs, and demonstrated a close relationship based on SNP analyses (0 to 7 SNP differences; Data Set S2). Analysis of the 50 calibration isolates using the novel E. faecalis cgMLST scheme confirmed the close relationship of those strains (Fig. S2A). Based on these results, and considering highly related and nonrelated members of the strain collection, we defined a difference of seven alleles as the threshold for distinct clusters.

### Concordance of the cgMLST scheme with SNP-based phylogenetic analyses.

To evaluate the newly defined cgMLST scheme, the results obtained were compared with an SNP-based mapping approach. A total of 2,200 SNP positions (recombinatoric events excluded by distance-based filtering) were considered for tree calculation. The resulting maximum likelihood phylogenetic tree had 147 tips (including reference genome) with 292 nodes, and it demonstrated that the isolates differed from each other by 0 to 213 SNPs (Fig. S3A; Data Set S2). The cgMLST-based phylogenetic tree consisted of 146 tips and 291 nodes, roughly indicating comparable outcomes (Fig. S3B). Both methods for phylogenetic tree calculation showed concordant clustering according to ST grouping and that of isolate clusters (>2 isolates) comprising several STs (Fig. S3).

For visualization and as a measure of concordance, a heat map was created, which was calculated based on the normalized absolute patristic distances for each particular isolate obtained by the two different approaches (Fig. S4). The heat map comparison showed a good concordance for closely related strains, such as the ST6 subpopulation, and for the groups of the ICS program (Fig. S4, visualized by blue boxes). The more distantly related isolates performed differently with the two approaches (Fig. S4, visualized by orange boxes).

### Application of the novel cgMLST scheme to describe the E. faecalis population structure.

**FIG 3:**
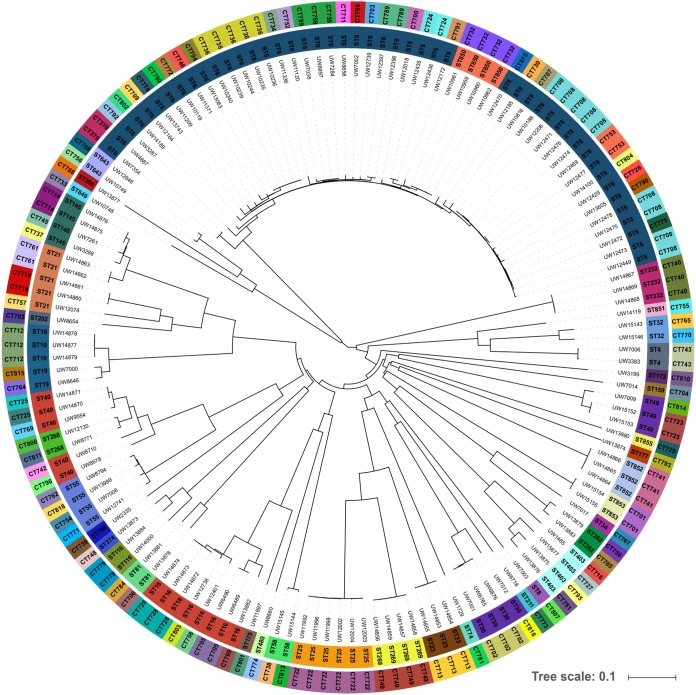
Isolate collection for establishing the E. faecalis cgMLST scheme (*n* = 146). The phylogenetic tree is based on cgMLST analyses and was calculated with SeqSphere^+^. Available data for sequence types (STs; inner circle) and complex types (CTs; outer circle) are depicted as colored circles. The collection comprised 41 different STs and 97 different CTs. Visualization was realized using iTOL.

The cgMLST scheme was also tested to investigate the relatedness of strains putatively involved in hospital outbreak events and to obtain further insights into E. faecalis population structure. Over 95% good cgMLST target genes were found in all isolate sequences of the entire strain collection, including those that were not used to define the cgMLST scheme (mean, 99.7% good target genes). The allocation of complex types (CTs) showed that the collection was composed of 97 different CTs (Fig. 3). To further visualize the results, an MST was calculated (Fig. S2). The isolates that were chosen for CT calibration were clearly represented by distinct clusters in the MST analysis (Fig. S2A). Due to the epidemiological data and previous PFGE analyses, PHT group 1 was identified, and indeed showed a maximum difference of six alleles in the cgMLST analysis. The isolates belonged to ST25 and CT722. The NCBI isolate E. faecalis LD33, which is also ST25 but exhibits CT237, was unequivocally separated from PHT group 1 by 490 alleles. Furthermore, isolates belonging to PHT groups 2 and 3 displayed identical CTs within each cluster and thus also proved suitable for complex calibration (0- to 2-allele difference). In contrast, PHT group 4 comprises eight ST6 isolates that were also demonstrated to be closely related in previous PFGE analyses (>85% identity; Fig. S1) and in SNP-based examination (0 to 11 SNPs; Data Set S2) but yielded four different CTs (0 to 15-allele difference; Fig. S2B).

The calibration collection also included 27 isolates sampled as part of an ICS program of healthy families in Germany (Fig. S2A). Previous PFGE analyses revealed that these isolates belonged to 10 distinct groups, with each screened family creating a distinct group (data not shown). The cgMLST analyses (>99.6% good target genes) generated results concordant with PFGE typing. The ICS groups generated no separate cluster of commensal isolates within the population, and instead these isolates were widely distributed over the MST (Fig. S2). Altogether, the ICS groups were suitable for calibration, as they differed by at most two alleles from each other within one group, but by over 29 alleles in relation to unrelated isolates harboring identical STs.

In addition, a selection of isolates that were not used to define the cgMLST scheme was analyzed. These isolates included 14 clinical isolates and also 21 environmental samples of E. faecalis. With a mean of 99.4% good target genes, all environmental isolates were successfully analyzed. Six isolates belonged to ST6 and were obtained directly from clinical sewage. Concordantly, those isolates clustered together with ST6 isolates of the strain collection in MST analyses (Fig. S2B). Nevertheless, they constituted a distinct cluster when considering the corresponding distance matrix (>27-allele difference from other ST6 isolates) and exhibited CT744. The other environmental isolates were distributed over the tree, harbored unique CTs, and showed no distinct clustering depending on the sampling site (Fig. S2A).

The 14 clinical isolates that were obtained from blood culture and sent to the NRC in the years 2015 to 2017 yielded 11 different STs and only unique CTs (mean, 99.4% good target genes). Four isolates were assigned to ST6 and also clustered within the ST6 group, but in contrast to the sewage, ST6 isolates did not separate into a distinct clade (Fig. S2B).

## DISCUSSION

In this study, we developed and evaluated a robust typing scheme for analyzing E. faecalis, an opportunistic pathogen of various origins. The scheme was implemented in the Ridom SeqSphere^+^ software suite to generate highly concordant and comparable results and to provide a standardized nomenclature for the scientific community.

The E. faecalis strain OG1RF was used as the seed genome to define the cgMLST targets. Based on previous investigations of comparative genomics, the OG1RF strain represents a rather conserved E. faecalis genome due to the lack of mobile genetic elements (44). In contrast, and thus less suitably for a cgMLST definition, the fully sequenced E. faecalis strain V583 was demonstrated to contain up to 25% mobile genetic elements within its genome (18).

The novel cgMLST scheme proved to be a powerful tool for analyzing E. faecalis isolates, including those of various sampling backgrounds, such as hospital-associated strains and strains obtained from general screenings in outpatient settings (colonization), but also for strains from animals, food, and the environment. Furthermore, the results of the evaluation with genome sequences from the NCBI and SRA databases highlight the robustness of the selected target genes and thus the suitability of the scheme to investigate the relatedness of E. faecalis isolates based on core genome data.

The results have also provided a detailed overview that enhances our current and common understanding of the E. faecalis population structure. When the classical MLST scheme for E. faecalis was published, the first insights into the population structure of this opportunistic pathogen became available (23). The authors described correlating results obtained by MLST and PFGE, but they also mentioned slight deviations between the two typing methods and additionally noted the influence of mobile genetic elements and high rates of recombination events. In accordance with the results of the cgMLST analyses in the present study, a dispersion of single genotypes over various origins and sampling sites was reported. However, a few lineages (e.g., ST6) showed adaptation to the hospital environment, which is especially known for E. faecium clinical isolates (21, 45–47).

The 14 isolates from blood cultures that were received by the NRC between 2015 and 2017 are represented by 11 different sequence types and 14 different complex types. This again supports the hypothesis that there is no distinct lineage responsible for human infections. Nonetheless, four of the isolates belonged to ST6, which suggests that at least some STs might primarily be human associated (21, 23). These results are in accordance with ST6, which is detected with a high prevalence in hospitalized patients (23, 48), as a human-associated lineage. Tedim et al. attributed the high prevalence of ST6 (and other highly abundant STs) to their high potential for adaptation (45). To create a robust scheme for E. faecalis outbreak analysis, we therefore included a high number (*n* = 47; 32%) of ST6 isolates in our strain collection used for scheme definition. The ST6 isolates incorporated into this study were collected over 18 years and originated exclusively from humans, including from the most common clinical presentations, such as bacteremia and endocarditis. In total, the ST6 strains analyzed comprised 57 isolates that yielded 34 different complex types. As was anticipated prior to setting up the cgMLST scheme, subdifferentiation was able to reveal multiple CTs within a certain ST, and thus might be suitable for tracing or excluding transmission events.

One considerable advantage of cgMLST, especially for genotyping purposes, is the focus on alleles instead of SNPs. While a missing gene or homologous recombination events can result in numerous SNPs, only entire allele changes are taken into account by cgMLST analyses (31, 49). In addition, the threshold of CT definition mitigates possible typing variations caused by these events. Indeed, cgMLST provides a robust and expandable database for allelic profiles, and it has already been applied for outbreak analyses of various pathogenic bacteria, such as Enterococcus faecium, Staphylococcus aureus, Listeria monocytogenes, and Clostridium difficile (29, 33, 34, 50).

Besides cgMLST, which is superior in its resolution compared to classical typing methods, reference-based bioinformatics approaches are widely used for more detailed analysis of strain relatedness and for phylogenetic purposes (51). Such approaches provide results in high resolution for investigating closely related individuals, as in outbreaks mostly restricted to a local setting (e.g., one ward, one hospital, or one country) (52). In contrast, phylogenetic investigations on a “global” scale or long-term studies often lack a reference suitable for the entire set of strains to be analyzed (53). Although programs such as refRank will select for the best reference for a given data set, it is always a trade-off with respect to the genomic variety of the strain collection (39). In addition, SNP-based approaches must be adjusted for recombination events, which would otherwise impact phylogenetic interpretations (49, 54).

Assessing the phylogenetic trees of the two approaches, the phylogenies seemed to be conformable and reproducible, as described before for comparative analyses of L. monocytogenes (55). Also, our mathematical approach to comparing the concordance of patristic distances obtained by SNP-based and cgMLST analyses demonstrated that a high concordance could be reached for closely related isolates. Isolates without a close relationship showed less concordant results. Taking into consideration that the SNP-based phylogenetic approach also includes intergenic regions, while cgMLST does not, the low concordance observed for distantly related isolates is expected.

We also analyzed and inversely examined the concordance of the cgMLST and SNP-based approaches using our strain collection. For instance, isolates of one of the putative hospital transmission groups (PHT group 2) were proven to be closely related both by SNP-based mapping and by cgMLST data. Analyses of the PHT group 2 and 3 strains, which originated from the same hospital and ward, were isolated 6 months apart and harbored different STs and CTs, a finding that was also supported by the SNP-based approach (7 SNPs). For isolates of PHT group 4, the cgMLST analyses showed that these strains belonged to three different CTs, revealing that this group was not built by the spread of a single clone. These findings again indicate the general applicability of cgMLST. Furthermore, it highlights the need to select a suitable reference genome for downstream analyses of variant-calling investigations in order to produce reliable results, by applying the most reasonable method for data evaluation at a given time and for a certain strain collection.

In conclusion, we successfully defined a cgMLST scheme for E. faecalis. Our analyses showed that cgMLST was suitable for analyzing closely related strains from putative hospital transmission events, as well as for analyzing and portraying the general population structure of this important opportunistic pathogen.

## Supplementary Material

Supplemental file 1

Supplemental file 2

Supplemental file 3

Supplemental file 4

Supplemental file 5

Supplemental file 6

Supplemental file 7
