# Supplementary material for: A Core Genome Multilocus Sequence Typing Scheme for Enterococcus faecalis
Source: J Clin Microbiol. 2019 Feb 27;57(3):e01686-18. doi: 10.1128/JCM.01686-18 (PMC6425188; doi:10.1128/JCM.01686-18)
Supplement: Supplemental file 3 [file JCM.01686-18-s0003.pdf]

FIG S1

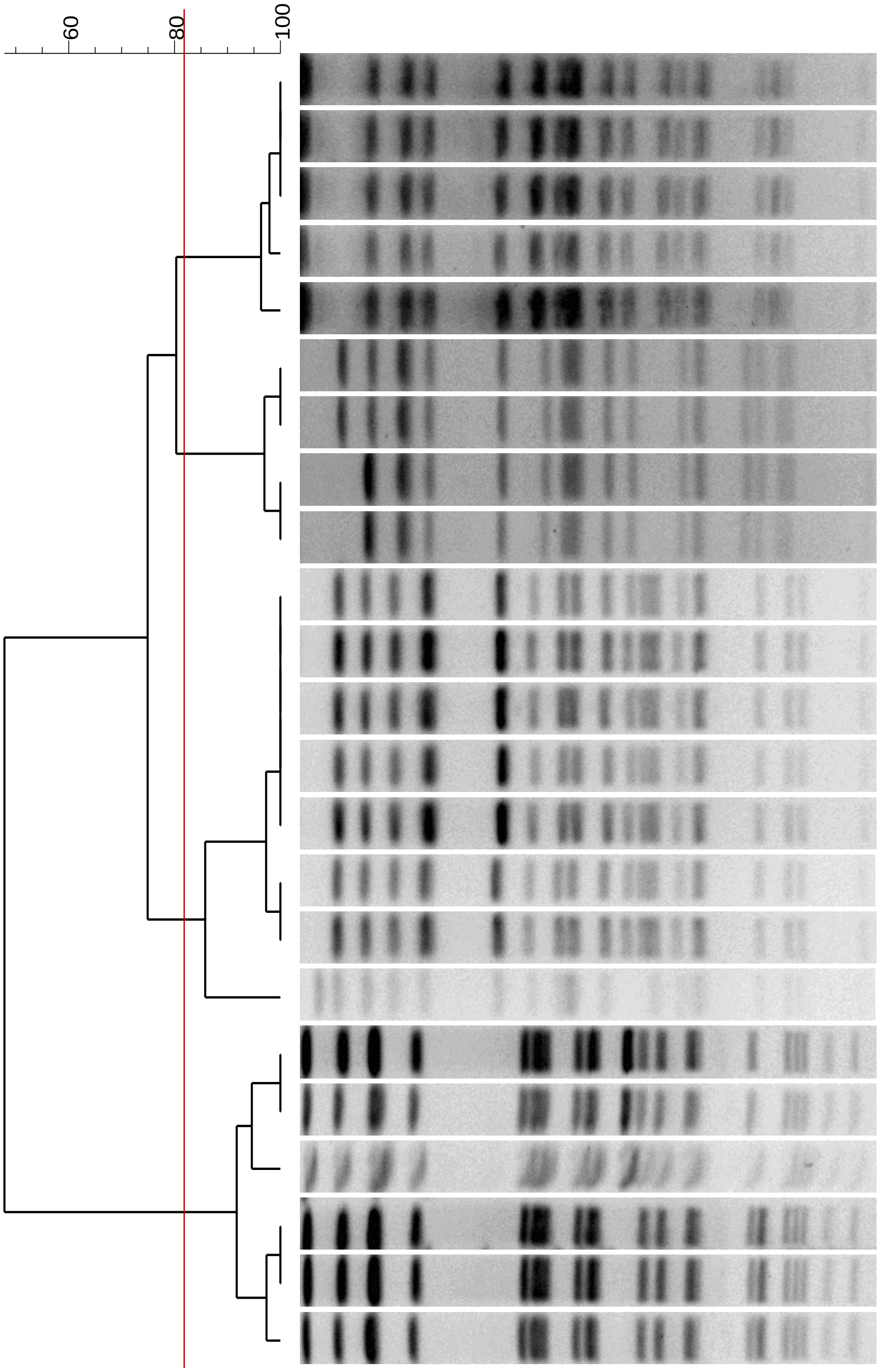

Strain ID	Sequence Type (ST)	Complex Type (CT)	
UW10236	ST6	CT736	group 3
UW10239	ST6	CT736	group 3
UW10240	ST6	CT736	group 3
UW10244	ST6	CT736	group 3
UW10235	ST6	CT736	group 3
UW10960	ST850	CT732	group 2
UW10961	ST850	CT732	group 2
UW10959	ST850	CT732	group 2
UW10962	ST850	CT732	group 2
UW12474	ST6	CT708	group 4
UW12475	ST6	CT708	group 4
UW12476	ST6	CT708	group 4
UW12477	ST6	CT753	group 4
UW12478	ST6	CT708	group 4
UW12469	ST6	CT753	group 4
UW12472	ST6	CT775	group 4
UW12470	ST6	CT812	group 4
UW11992	ST25	CT722	group 1
UW12003	ST25	CT722	group 1
UW12004	ST25	CT722	group 1
UW11996	ST25	CT722	group 1
UW11998	ST25	CT722	group 1
UW12002	ST25	CT722	group 1

FIG S1 *Sma*I-macrorestriction analysis of genomic DNA and subsequent pulsed-field gel electrophoresis (PFGE) of 23 *E. faecalis* isolates designated to define complex types (CT). The DNA band patterns and the dendrogram show the close relationship of the isolates. The red line delineates the similarity value of 82 % for grouping. The four groups (>82 % identity) belonged to four putative hospital transmission events (PHT). The visualization (including dendrogram) was realized using BioNumerics.
