# Supplementary material for: A Core Genome Multilocus Sequence Typing Scheme for Enterococcus faecalis
Source: J Clin Microbiol. 2019 Feb 27;57(3):e01686-18. doi: 10.1128/JCM.01686-18 (PMC6425188; doi:10.1128/JCM.01686-18)
Supplement: Supplemental file 4 [file JCM.01686-18-s0004.pdf]

FIG S2

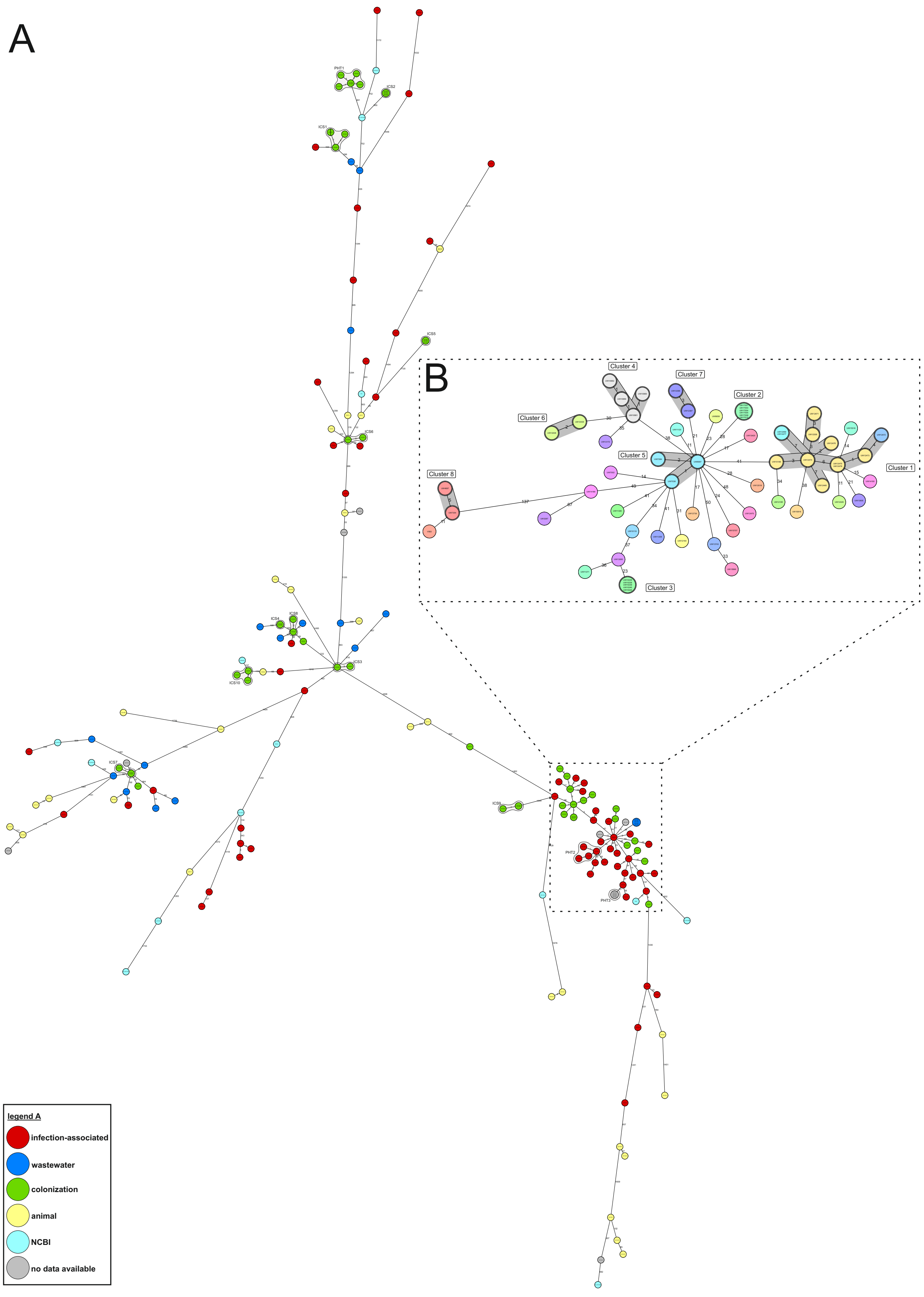
A
B
PHT1
ICS1
ICS2
ICS3
ICS4
ICS5
ICS6
ICS7
ICS8
ICS10
PHT2
PHT3
Cluster 1
Cluster 2
Cluster 3
Cluster 4
Cluster 5
Cluster 6
Cluster 7
Cluster 8
legend A
infection-associated
wastewater
colonization
animal
NCBI
no data available

FIG S2 Minimum spanning tree (MST) based on allelic profiles determined by cgMLST analysis using the SeqSphere+ software of 195 *E. faecalis* isolates, also including all available completed genomes from NCBI (upon request). Each circle represents an *E. faecalis* strain analyzed by the typing scheme. The number on the connecting lines represents the alleles difference between two isolates. (A) The coloring according to sample origin (see legend) shows no distinct clustering of single isolates, except for the known association of ST6 isolates to human origin. The isolate groups (intestinal screening study (ICS); putative hospital transmissions (PHT)) used for cluster calibration are shaded in gray. (B) A sub-MST of the 58 strains with ST6. The color code depicts individual complex types (CT). Isolates are shaded in gray in case the allele difference is 7 or less and thus represent distinct clusters. As for example, cluster 1 represents isolates of PHT group 4. Within PHT group 4, isolate UW12206 (colored in blue) is closely related to PHT group 4 (same hospital, same CT) but comprises a different CT. This cluster also comprises related isolates showing an epidemiological connection (same hospital, same time) but which were previously not defined as part of PHT group 4.
