# Supplementary material for: A Core Genome Multilocus Sequence Typing Scheme for Enterococcus faecalis
Source: J Clin Microbiol. 2019 Feb 27;57(3):e01686-18. doi: 10.1128/JCM.01686-18 (PMC6425188; doi:10.1128/JCM.01686-18)
Supplement: Supplemental file 5 [file JCM.01686-18-s0005.pdf]

FIG S3

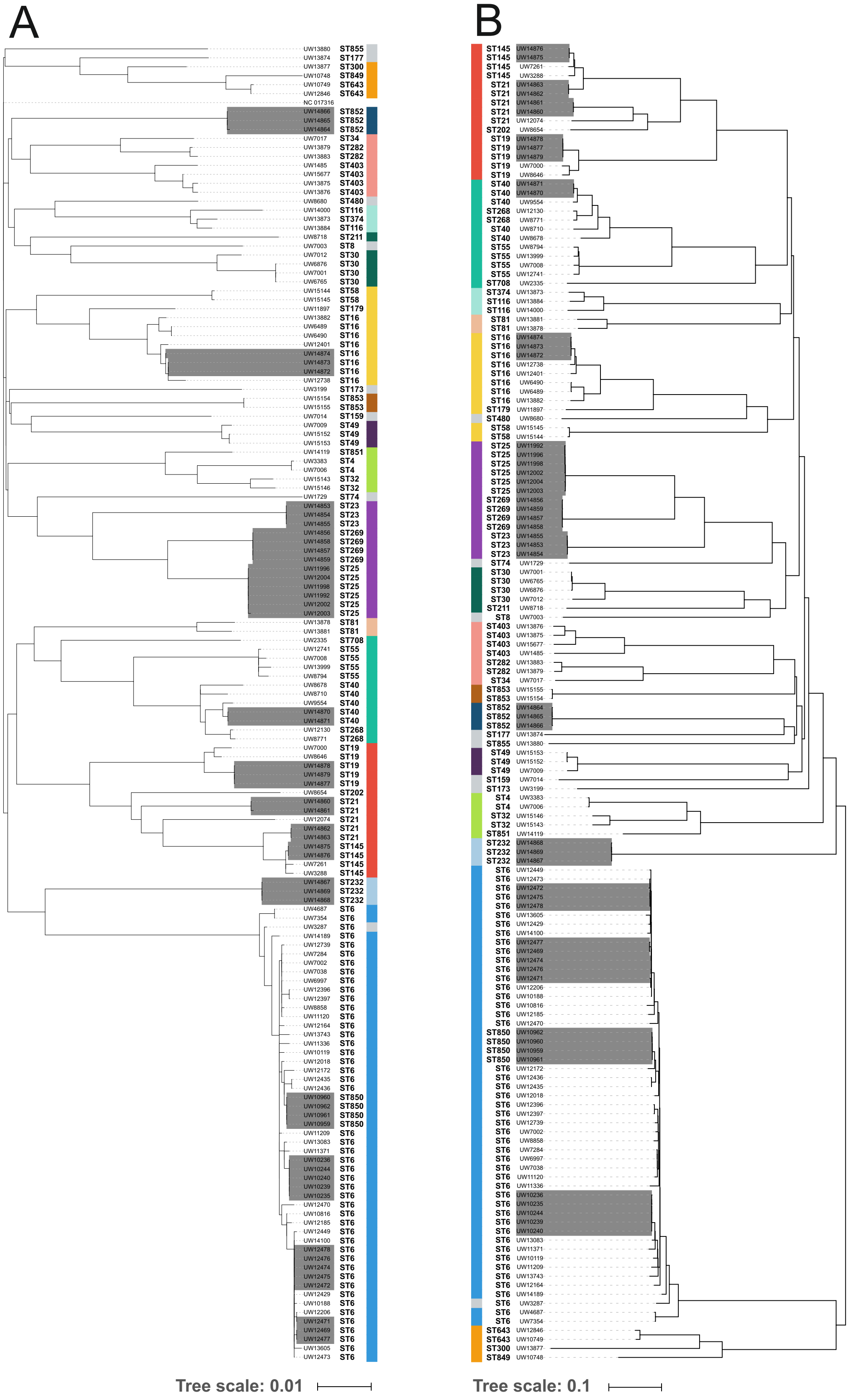

FIG S3 Comparison of the (A) SNP-based and (B) the cgMLST-based phylogenetic tree of the 146 isolates used for scheme definition. Strain ID and sequence types (ST) are displayed. Color coding depicts clusters of isolate groups (>2 isolates). 15 clusters could be identified in both phylogenetic trees. The light blue color represents the distinct ST6/ST850 cluster. Light gray color coded isolates represent Singletons. The OG1RF strain was used as reference for the SNP-based phylogenetic tree (no color assigned; A). Isolate groups that were included for complex type calibration (see Table S1 “definition/ calibration”) were shaded in dark gray (A, B). Visualization was realized using iTOL.
