# Supplementary material for: A Core Genome Multilocus Sequence Typing Scheme for Enterococcus faecalis
Source: J Clin Microbiol. 2019 Feb 27;57(3):e01686-18. doi: 10.1128/JCM.01686-18 (PMC6425188; doi:10.1128/JCM.01686-18)
Supplement: Supplemental file 6 [file JCM.01686-18-s0006.pdf]

FIG S4

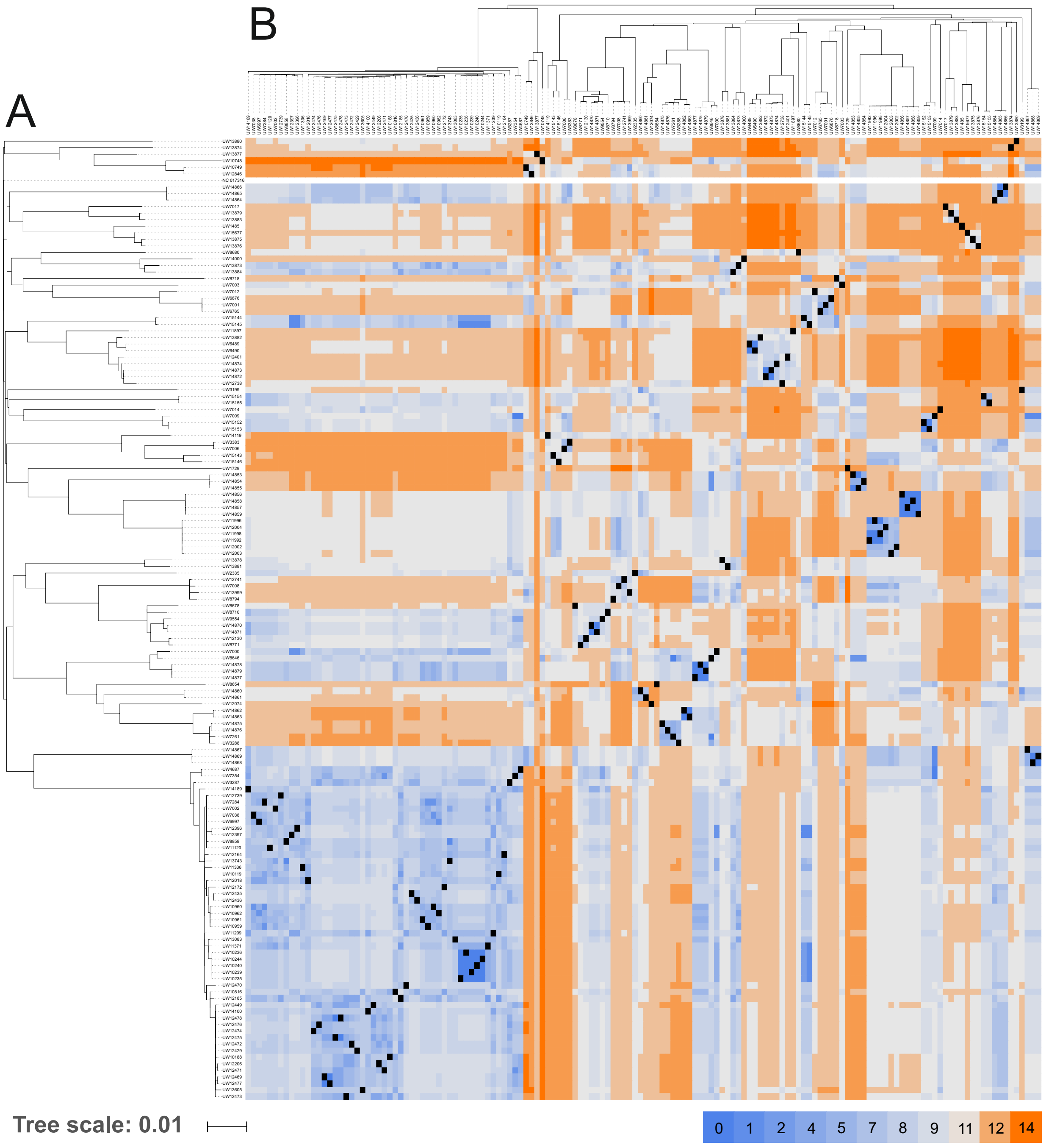

**FIG S4** Visualization of the comparison of the phylogenetic results obtained by a (A) SNP-based approach (mapping and variant calling) and by (B) cgMLST analysis. The color codes of the heatmap display the absolute difference (the distance between two leaves measured along the tree) of isolate pairs obtained by the respective phylogenetic analyses. Small differences in distances (“0”) are shown in blue. Large differences in distances (“15”) are shown in orange. To lessen the effect of logarithmic data distribution on the visualization, the median of the values was calculated and used as the mean value of the color scale. The color gray represents the median of all distance differences (“11”). Visualization was realized using the web-tool iTOL.
