# Supplementary material for: A Core Genome Multilocus Sequence Typing Scheme for Enterococcus faecalis
Source: J Clin Microbiol. 2019 Feb 27;57(3):e01686-18. doi: 10.1128/JCM.01686-18 (PMC6425188; doi:10.1128/JCM.01686-18)
Supplement: Supplemental file 7 [file JCM.01686-18-s0007.pdf]

Table S1: List of *E. faecalis* isolates/genome sequences used for cgMLST target definition and population analysis.

Strain ID	State of Isolation [a)]	Year of Isolation [b)]	Source [c)]	Isolation Site [d)]	Sampling	Sequence Type	Complex Type	*van-genotype*	% good cgMLST targets	Note	Reference
OG1RF	USA	2011	H/L	n.d.	n.d.	1	1	none	100	reference/ seed genome	NC_017316.1
V583	USA	2003	H/L	n.d.	n.d.	6	371	*vanB*	99.4	definition/ NCBI	NC_004668.1
Symbioflor1	Germany	2012	P/L	n.d.	n.d.	248	211	none	99.5	definition/ NCBI	NC_019770.1
D32	Denmark	2012	A	S	n.d.	40	146	none	99.1	definition/ NCBI	NC_018221.1
DENG1	China	2014	H	N	n.d.	191	152	n.d.	99.6	definition/ NCBI	NZ_CP004081.1
L12	Brazil	2016	A	S	n.d.	711	235	n.d.	99.9	definition/ NCBI	NZ_CP018102.1
LD33	China	2016	F	n.d.	n.d.	25	237	n.d.	99.6	definition/ NCBI	NZ_CP014949.1
ATCC29212	USA	2014	L	n.d.	n.d.	30	104	n.d.	98.1	definition/ NCBI	NZ_CP008816.1
KB1	Switzerland	2016	A	M	n.d.	9	259	n.d.	97.1	definition/ NCBI	NZ_CP015410.2
W11	Japan	2016	F	n.d.	n.d.	u	809	n.d.	99.8	definition/ NCBI	NZ_AP017623.1
L9	Brazil	2016	A	S	n.d.	29	261	n.d.	99.7	definition/ NCBI	NZ_CP018004.1
CLB21560	USA	2017	H	N	n.d.	28	262	n.d.	99.6	definition/ NCBI	NZ_CP019512.1
UW1485	Saxony-Anhalt	1997	H	N	unknown	403	716	*vanA*	99.8	definition	this study
UW1729	Baden-Wuerttemberg	1998	H	N	infection associated	74	781	none	98.8	definition	this study
UW2335	Hamburg	1999	H	N	infection associated	708	739	*vanA*	99.7	definition	this study
UW3199	Berlin	2001	H	N	infection associated	173	810	none	99.4	definition	this study
UW3287	Berlin	2001	H	N	infection associated	6	792	none	99.7	definition	this study
UW3288	Berlin	2001	H	N	infection associated	145	737	none	99.7	definition	this study
UW3383	Berlin	2001	H	N	infection associated	4	743	none	99.5	definition	this study
UW4687	Berlin	2004	H	N	colonization	6	370	*vanB*	99.6	definition	this study
UW6489	Berlin	2006	H	N	infection associated	16	705	*vanA*	99.8	definition	this study
UW6490	Berlin	2006	H	N	infection associated	16	705	*vanA*	99.8	definition	this study
UW6765	North Rhine-Westphalia	2006	H	N	infection associated	30	702	none	99.2	definition	this study
UW6876	Mecklenburg-West Pomerania	2007	H	N	infection associated	30	702	*vanB*	99.1	definition	this study
UW6997	Mecklenburg-West Pomerania	2007	H	N	infection associated	6	759	*vanB*	99.9	definition	this study
UW7000	Mecklenburg-West Pomerania	2007	H	N	infection associated	19	815	none	99.6	definition	this study
UW7001	Mecklenburg-West Pomerania	2007	H	N	infection associated	30	702	none	99.2	definition	this study
UW7002	Mecklenburg-West Pomerania	2007	H	N	infection associated	6	798	none	99.9	definition	this study
UW7003	Mecklenburg-West Pomerania	2007	H	N	infection associated	8	807	none	99.3	definition	this study
UW7006	Mecklenburg-West Pomerania	2007	H	N	infection associated	4	743	none	99.4	definition	this study
UW7008	Mecklenburg-West Pomerania	2007	H	N	infection associated	55	754	none	99.5	definition	this study
UW7009	Mecklenburg-West Pomerania	2007	H	N	infection associated	49	814	none	99.7	definition	this study
UW7012	Baden-Wuerttemberg	2007	H	N	infection associated	30	816	none	99.6	definition	this study
UW7014	Baden-Wuerttemberg	2007	H	N	infection associated	159	704	none	99.3	definition	this study
UW7017	Baden-Wuerttemberg	2007	H	N	infection associated	34	767	none	99.3	definition	this study
UW7038	Mecklenburg-West Pomerania	2007	H	N	infection associated	6	759	*vanB*	99.9	definition	this study
UW7261	Thuringia	2007	H	N	colonization	145	745	*vanA*	99.8	definition	this study
UW7284	Mecklenburg-West Pomerania	2007	H	N	infection associated	6	759	*vanB*	99.8	definition	this study
UW7354	Portugal	2008	H	N	infection associated	6	370	*vanB*	99.8	definition	this study
UW8646	Lower Saxony	2011	A	D	mastitis	19	764	none	99.7	definition	*1
UW8654	Lower Saxony	2011	A	D	mastitis	202	763	none	99.8	definition	*1
UW8678	Lower Saxony	2011	A	D	mastitis	40	796	none	99.7	definition	*1
UW8680	Lower Saxony	2011	A	D	mastitis	480	774	none	99.6	definition	*1
UW8710	Lower Saxony	2011	A	D	mastitis	40	742	none	99.6	definition	*1
UW8718	Lower Saxony	2011	A	D	mastitis	211	773	none	99.3	definition	*1
UW8771	Lower Saxony	2011	A	D	mastitis	268	811	none	99.6	definition	*1
UW8794	Lower Saxony	2011	A	D	mastitis	55	762	none	99.7	definition	*1
UW8858	Berlin	2011	H	N	infection associated	6	711	*vanB*	99.8	definition	this study
UW9554	Bavaria	2012	H	N	infection associated	40	769	*vanB*	99.6	definition	this study
UW10119	Baden-Wuerttemberg	2012	H	N	infection associated	6	772	none	99.8	definition	this study
UW10188	Netherlands	2012	H	N	infection associated	6	708	*vanB*	100.0	definition	this study
UW10235	Lower Saxony	2012	H	N	unknown	6	736	none	100.0	definition/ calibration	this study
UW10236	Lower Saxony	2012	H	N	unknown	6	736	none	100.0	definition/ calibration	this study
UW10239	Lower Saxony	2012	H	N	unknown	6	736	none	99.9	definition/ calibration	this study
UW10240	Lower Saxony	2012	H	N	unknown	6	736	none	99.9	definition/ calibration	this study
UW10244	Lower Saxony	2012	H	N	unknown	6	736	none	100.0	definition/ calibration	this study
UW10748	Ireland	2013	H	N	infection associated	849	733	*vanA*	98.8	definition	this study
UW10749	Ireland	2013	H	N	infection associated	643	756	*vanA*	99.0	definition	this study
UW10816	Saxony	2013	H	N	infection associated	6	707	none	96.4	definition	this study
UW10959	Lower Saxony	2013	H	N	infection associated	850	732	none	99.9	definition/ calibration	this study
UW10960	Lower Saxony	2013	H	N	infection associated	850	732	none	99.9	definition/ calibration	this study
UW10961	Lower Saxony	2013	H	N	infection associated	850	732	none	99.9	definition/ calibration	this study
UW10962	Lower Saxony	2013	H	N	infection associated	850	732	none	99.9	definition/ calibration	this study
UW11120	Mecklenburg-West Pomerania	2013	H	N	unknown	6	752	*vanB*	97.7	definition	this study
UW11209	Saxony	2013	H	N	colonization	6	780	*vanA*	99.7	definition	this study
UW11336	Bavaria	2013	H	N	colonization	6	734	*vanA*	99.7	definition	this study
UW11371	North Rhine-Westphalia	2013	H	N	infection associated	6	746	*vanA*	99.8	definition	this study
UW11897	Saxony	2014	H	N	infection associated	179	801	*vanA*	99.7	definition	this study
UW11992	Lower Saxony	2014	H	N	colonization	25	722	none	99.8	definition/ calibration	this study
UW11996	Lower Saxony	2014	H	N	colonization	25	722	none	99.8	definition/ calibration	this study
UW11998	Lower Saxony	2014	H	N	colonization	25	722	none	99.8	definition/ calibration	this study
UW12002	Lower Saxony	2014	H	N	colonization	25	722	none	99.8	definition/ calibration	this study
UW12003	Lower Saxony	2014	H	N	colonization	25	722	none	99.3	definition/ calibration	this study
UW12004	Lower Saxony	2014	H	N	colonization	25	722	none	99.8	definition/ calibration	this study
UW12018	Bavaria	2014	H	N	infection associated	6	700	*vanB*	99.8	definition	this study

UW12074	Saxony-Anhalt	2014	H	N	infection associated	21	757	none	99.8	definition	this study
UW12130	Saxony-Anhalt	2014	H	N	infection associated	268	808	none	99.3	definition	this study
UW12164	Baden-Wuerttemberg	2014	H	N	infection associated	6	709	none	99.9	definition	this study
UW12172	Baden-Wuerttemberg	2014	H	N	infection associated	6	791	none	99.8	definition	this study
UW12185	Berlin	2014	H	N	infection associated	6	730	none	99.7	definition	this study
UW12206	Saxony	2014	H	N	colonization	6	708	*vanB*	99.9	definition	this study
UW12396	Berlin	2014	H	N	colonization	6	789	*vanA*	99.7	definition	this study
UW12397	Berlin	2014	H	N	colonization	6	789	*vanA*	99.7	definition	this study
UW12401	Berlin	2014	H	N	colonization	16	758	none	99.8	definition	this study
UW12429	Berlin	2014	H	N	infection associated	6	726	*vanB*	100.0	definition	this study
UW12435	Lower Saxony	2014	H	N	infection associated	6	724	none	99.7	definition	this study
UW12436	Lower Saxony	2014	H	N	infection associated	6	724	none	99.8	definition	this study
UW12449	Brandenburg	2015	H	N	colonization	6	708	*vanB*	99.8	definition	this study
UW12469	Saxony	2015	H	N	colonization	6	753	*vanA*	100.0	definition/ calibration	this study
UW12470	Saxony	2015	H	N	colonization	6	812	*vanA*	99.9	definition	this study
UW12471	Saxony	2015	H	N	colonization	6	708	*vanA*	100.0	definition/ calibration	this study
UW12472	Saxony	2015	H	N	colonization	6	775	*vanA*	100.0	definition/ calibration	this study
UW12473	Saxony	2015	H	N	colonization	6	708	*vanB*	100.0	definition	this study
UW12474	Saxony	2015	H	N	colonization	6	708	*vanA*	99.9	definition/ calibration	this study
UW12475	Saxony	2015	H	N	colonization	6	708	*vanA*	100.0	definition/ calibration	this study
UW12476	Saxony	2015	H	N	colonization	6	708	*vanA*	99.9	definition/ calibration	this study
UW12477	Saxony	2015	H	N	colonization	6	753	*vanA*	100.0	definition/ calibration	this study
UW12478	Saxony	2015	H	N	colonization	6	708	*vanA*	100.0	definition/ calibration	this study
UW12738	Berlin	2015	H	N	unknown	16	803	none	99.7	definition	this study
UW12739	Berlin	2015	H	N	unknown	6	703	none	99.9	definition	this study
UW12741	Berlin	2015	H	N	unknown	55	771	none	99.6	definition	this study
UW12846	Ireland	2015	H	N	infection associated	643	768	*vanA*	99.0	definition	this study
UW13083	North Rhine-Westphalia	2015	H	N	infection associated	6	794	*vanA*	99.9	definition	this study
UW13605	Brandenburg	2015	H	N	infection associated	6	790	*vanB*	98.6	definition	this study
UW13743	Bavaria	2015	H	N	colonization	6	776	*vanB*	99.6	definition	this study
UW13873	Mecklenburg-West Pomerania	2015	A	P	meat	374	748	none	99.7	definition	this study
UW13874	Mecklenburg-West Pomerania	2015	A	P	meat	177	793	none	99.1	definition	this study
UW13875	Mecklenburg-West Pomerania	2015	A	P	meat	403	795	none	99.9	definition	this study
UW13876	Mecklenburg-West Pomerania	2015	A	P	meat	403	751	none	99.8	definition	this study
UW13877	Mecklenburg-West Pomerania	2015	A	P	meat	300	788	none	98.3	definition	this study
UW13878	Mecklenburg-West Pomerania	2015	A	P	meat	81	706	none	99.4	definition	this study
UW13879	Mecklenburg-West Pomerania	2015	A	P	meat	282	750	none	99.3	definition	this study
UW13880	Mecklenburg-West Pomerania	2015	A	P	meat	855	729	none	99.6	definition	this study
UW13881	Mecklenburg-West Pomerania	2015	A	P	meat	81	784	none	99.5	definition	this study
UW13882	Mecklenburg-West Pomerania	2015	A	P	meat	16	799	none	99.9	definition	this study
UW13883	Mecklenburg-West Pomerania	2015	A	P	meat	282	785	none	99.3	definition	this study
UW13884	Mecklenburg-West Pomerania	2015	A	P	meat	116	779	none	99.3	definition	this study
UW13999	North Rhine-Westphalia	2016	H	N	unknown	55	818	none	99.2	definition	this study
UW14000	North Rhine-Westphalia	2016	H	N	unknown	116	779	none	99.5	definition	this study
UW14100	Brandenburg	2016	H	N	infection associated	6	804	*vanB*	98.8	definition	this study
UW14119	Bavaria	2016	H	N	colonization	851	755	*vanB*	99.7	definition	this study
UW14189	North Rhine-Westphalia	1999	H	N	infection associated	6	802	none	99.9	definition	this study
UW14853	Baden-Wuerttemberg	2005	H	C	colonization	23	713	none	99.7	definition/ calibration	this study
UW14854	Baden-Wuerttemberg	2005	H	C	colonization	23	713	none	99.7	definition/ calibration	this study
UW14855	Baden-Wuerttemberg	2005	H	C	colonization	23	713	none	99.7	definition/ calibration	this study
UW14856	Baden-Wuerttemberg	2005	H	C	colonization	269	749	none	99.9	definition/ calibration	this study
UW14857	Baden-Wuerttemberg	2005	H	C	colonization	269	749	none	99.9	definition/ calibration	this study
UW14858	Baden-Wuerttemberg	2005	H	C	colonization	269	749	none	99.9	definition/ calibration	this study
UW14859	Baden-Wuerttemberg	2005	H	C	colonization	269	749	none	99.9	definition/ calibration	this study
UW14860	Baden-Wuerttemberg	2005	H	C	colonization	21	718	none	99.7	definition/ calibration	this study
UW14861	Baden-Wuerttemberg	2005	H	C	colonization	21	718	none	99.7	definition/ calibration	this study
UW14862	Baden-Wuerttemberg	2005	H	C	colonization	21	761	none	99.7	definition/ calibration	this study
UW14863	Baden-Wuerttemberg	2005	H	C	colonization	21	761	none	99.7	definition/ calibration	this study
UW14864	Baden-Wuerttemberg	2005	H	C	colonization	852	741	none	99.7	definition/ calibration	this study
UW14865	Baden-Wuerttemberg	2005	H	C	colonization	852	741	none	99.7	definition/ calibration	this study
UW14866	Baden-Wuerttemberg	2005	H	C	colonization	852	741	none	99.7	definition/ calibration	this study
UW14867	Baden-Wuerttemberg	2005	H	C	colonization	232	740	none	99.8	definition/ calibration	this study
UW14868	Baden-Wuerttemberg	2005	H	C	colonization	232	740	none	99.8	definition/ calibration	this study
UW14869	Baden-Wuerttemberg	2005	H	C	colonization	232	740	none	99.8	definition/ calibration	this study
UW14870	Baden-Wuerttemberg	2005	H	C	colonization	40	725	none	99.6	definition/ calibration	this study
UW14871	Baden-Wuerttemberg	2005	H	C	colonization	40	725	none	99.6	definition/ calibration	this study
UW14872	Baden-Wuerttemberg	2005	H	C	colonization	16	728	none	99.9	definition/ calibration	this study
UW14873	Baden-Wuerttemberg	2005	H	C	colonization	16	728	none	99.9	definition/ calibration	this study
UW14874	Baden-Wuerttemberg	2005	H	C	colonization	16	728	none	99.9	definition/ calibration	this study
UW14875	Baden-Wuerttemberg	2005	H	C	colonization	145	714	none	99.8	definition/ calibration	this study
UW14876	Baden-Wuerttemberg	2005	H	C	colonization	145	714	none	99.8	definition/ calibration	this study
UW14877	Baden-Wuerttemberg	2005	H	C	colonization	19	712	none	99.7	definition/ calibration	this study
UW14878	Baden-Wuerttemberg	2005	H	C	colonization	19	712	none	99.7	definition/ calibration	this study
UW14879	Baden-Wuerttemberg	2005	H	C	colonization	19	712	none	99.7	definition/ calibration	this study
UW15143	North Rhine-Westphalia	2016	A	S	feces	32	765	none	99.8	definition	this study
UW15144	North Rhine-Westphalia	2016	A	S	feces	58	813	none	99.7	definition	this study
UW15145	North Rhine-Westphalia	2016	A	S	feces	58	738	none	99.7	definition	this study
UW15146	North Rhine-Westphalia	2016	A	S	feces	32	770	none	99.9	definition	this study
UW15152	North Rhine-Westphalia	2016	A	S	feces	49	723	none	99.4	definition	this study
UW15153	North Rhine-Westphalia	2016	A	S	feces	49	723	none	99.4	definition	this study
UW15154	Hesse	2016	A	S	feces	853	701	none	99.6	definition	this study
UW15155	Hesse	2016	A	S	feces	853	701	none	99.6	definition	this study
UW15677	Lower Saxony	2016	A	S	feces	403	727	none	99.8	definition	this study
UW13420	Brandenburg	2015	H	N	infection associated	6	820	none	99.9	population analysis	this study

UW13959	Baden-Wuerttemberg	2015	H	N	infection associated	206	826	none	99.3	population analysis	this study
UW14781	Berlin	2016	H	N	infection associated	28	710	none	99.3	population analysis	this study
UW14828	Hesse	2016	H	N	infection associated	482	786	none	99.6	population analysis	this study
UW15202	Bavaria	2016	H	N	infection associated	26	735	none	99.7	population analysis	this study
UW15318	Brandenburg	2016	H	N	infection associated	6	747	*vanB*	98.8	population analysis	this study
UW15321	Berlin	2016	H	N	infection associated	59	806	none	99.8	population analysis	this study
UW15658	North Rhine-Westphalia	2016	H	N	infection associated	72	783	none	98.7	population analysis	this study
UW15707	Brandenburg	2016	H	N	infection associated	6	821	none	99.8	population analysis	this study
UW15842	Baden-Wuerttemberg	2017	H	N	infection associated	23	822	none	99.5	population analysis	this study
UW15896	North Rhine-Westphalia	2017	H	N	infection associated	6	819	*vanA*	99.3	population analysis	this study
UW16614	Berlin	2017	H	N	infection associated	8	823	none	99.4	population analysis	this study
UW16661	Brandenburg	2017	H	N	infection associated	40	824	none	99.4	population analysis	this study
UW16671	North Rhine-Westphalia	2017	H	N	infection associated	476	825	none	99.7	population analysis	this study
UW17001	Mecklenburg-West Pomerania	2017	E	U	wastewater	6	744	none	99.9	population analysis	this study
UW17003	Mecklenburg-West Pomerania	2017	E	U	wastewater	6	744	none	99.9	population analysis	this study
UW17004	Mecklenburg-West Pomerania	2017	E	U	wastewater	6	744	none	99.9	population analysis	this study
UW17005	Mecklenburg-West Pomerania	2017	E	U	wastewater	6	744	none	99.9	population analysis	this study
UW17008	Mecklenburg-West Pomerania	2017	E	U	wastewater	6	744	none	99.9	population analysis	this study
UW17015	Mecklenburg-West Pomerania	2017	E	U	wastewater	6	744	none	99.9	population analysis	this study
UW17034	Mecklenburg-West Pomerania	2017	E	U	wastewater	22	720	none	99.9	population analysis	this study
UW17045	Mecklenburg-West Pomerania	2017	E	U	wastewater	16	797	none	99.9	population analysis	this study
UW17049	Mecklenburg-West Pomerania	2017	E	U	wastewater	145	800	none	99.7	population analysis	this study
UW17002	Mecklenburg-West Pomerania	2017	E	U	wastewater	179	805	none	99.6	population analysis	this study
UW17017	Mecklenburg-West Pomerania	2017	E	T	wastewater	23	787	none	99.6	population analysis	this study
UW17030	Mecklenburg-West Pomerania	2017	E	T	wastewater	500	778	none	99.6	population analysis	this study
UW17009	Mecklenburg-West Pomerania	2017	E	T	wastewater	179	715	none	99.5	population analysis	this study
UW17022	Mecklenburg-West Pomerania	2017	E	T	wastewater	16	817	none	99.5	population analysis	this study
UW17027	Mecklenburg-West Pomerania	2017	E	T	wastewater	21	717	none	99.5	population analysis	this study
UW17040	Mecklenburg-West Pomerania	2017	E	T	wastewater	21	719	none	99.5	population analysis	this study
UW17044	Mecklenburg-West Pomerania	2017	E	T	wastewater	854	731	none	99.5	population analysis	this study
UW17018	Mecklenburg-West Pomerania	2017	E	T	wastewater	117	760	none	99.2	population analysis	this study
UW17043	Mecklenburg-West Pomerania	2017	E	T	wastewater	16	782	none	98.9	population analysis	this study
UW17024	Mecklenburg-West Pomerania	2017	E	T	wastewater	72	766	none	98.3	population analysis	this study
UW17042	Mecklenburg-West Pomerania	2017	E	T	wastewater	23	777	none	96.4	population analysis	this study

a) country of submitting institution for NCBI strains
b) year of release for NCBI strains
c) L=laboratory; H=human; A= animal; E= environment; F=food; P=probiotic
d) N= nosocomial; C= community; S= swine; P= poultry; D= dairy cattle; M=mouse; U= untreated wastewater; T= wastewater treatment
n.d.= no data available

Reference *1: Werner G, Fleige C, Fessler AT, Timke M, Kostrzewa M, Zischka M, Peters T, Kaspar H, Schwarz S. 2012. Improved identification including MALDI-TOF mass spectrometry analysis of group D streptococci from bovine mastitis and subsequent molecular characterization of corresponding *Enterococcus faecalis* and *Enterococcus faecium* isolates. Vet Microbiol 160:162-169.
